# Patient and Healthcare Provider Satisfaction with Sexual Assault Nurse Examiners (SANEs): A Systematic Review

**DOI:** 10.3390/healthcare12232399

**Published:** 2024-11-29

**Authors:** Alba Fernández-Collantes, Cristian Martín-Vázquez, María Cristina Martínez-Fernández

**Affiliations:** 1 Hospital El Bierzo, Gerencia de Asistencia Sanitaria del Bierzo, Servicio de Salud de Castilla y León, 24401 Ponferrada, Spain; albacollantess8@gmail.com; 2 Department of Nursing and Physiotherapy, Campus de Ponferrada, University of León, 24401 León, Spain; cmartv@unileon.es; 3 SALBIS Research Group, Faculty of Health Sciences, Campus de Ponferrada, University of León, 24401 León, Spain

**Keywords:** sexual assault, sexual assault nurse examiners, personal satisfaction, patient satisfaction

## Abstract

**Background/Objectives**: The World Health Organization (WHO) estimates that one in three women worldwide has experienced physical or sexual violence. In countries like the US, UK, and Canada, victims are often cared for by sexual assault nurse examiners (SANEs), who are trained to conduct forensic exams and offer emotional support, reducing the risk of retraumatisation. Thus, the aim of this study was to describe the satisfaction of patients and healthcare professionals with SANEs’ services. **Methods**: A systematic review was conducted by searching the PubMed, Web of Science, and Scopus databases, selecting studies that focused on patient and healthcare provider satisfaction with SANEs’ services. **Results**: In total, nine studies meeting the inclusion and exclusion criteria were analysed. Of these, 55% focused on healthcare provider satisfaction, while 44% examined the experiences of sexual assault survivors. All studies examining patient satisfaction with the care provided by SANE professionals (n = 3) reported satisfaction levels exceeding 90%, with many users highly recommending their services. **Conclusions**: The role of sexual assault nurse examiners is crucial in providing victims with a safe environment and quality of care, and in reducing the risk of retraumatisation.

## 1. Introduction

Sexual violence is a global, intergenerational problem that affects children, women, men, and non-binary individuals across all stages of life worldwide. The term encompasses any sexual act, attempt to commit a sexual act, unwanted sexual comments or advances, or actions to exploit or use a person’s sexuality in any other way [1]. There are numerous manifestations of sexual violence, including rape, sexual assault, sexual harassment, forced marriage, female genital mutilation, and forced prostitution [2].

The term “sexual assault” is defined as any unwanted sexual contact, including attacks such as rape or attempted rape, sodomy (oral or anal sexual acts), child sexual abuse, incest, or unwanted fondling [3]. In the majority of cases, the perpetrator is known to the victim or their family. However, in some instances, the perpetrator may be a stranger. To achieve their objective, the perpetrator may employ a range of tactics, including the use of violence, threats, coercion, manipulation, pressure, or deceit. Sexual assault is one of the most traumatic experiences a person can endure, affecting their physical, psychological (both short- and long-term), and social health [4].

The World Health Organization (WHO) has identified sexual violence as a significant global public health and human rights issue with far-reaching health consequences. Furthermore, it is estimated that between 2000 and 2018, 30% of women worldwide have experienced physical and/or sexual violence [5]. A substantial percentage of women aged 15 to 49 who have been in a relationship have experienced physical and/or sexual violence from an intimate partner at least once in their lives (since age 15). The lifetime prevalence of intimate partner violence varies, with estimates ranging from 20% in the Western Pacific, 22% in high-income countries and Europe, and 25% in the WHO region of the Americas, to 33% in the WHO African region, 31% in the WHO Eastern Mediterranean region, and 33% in the WHO South-East Asia region [6]. In Europe, approximately 9 million women over the age of 15 have been raped at some point in their lives, and approximately 3.7 million women experienced sexual violence in 2014 [7]. In 2017, the International Labour Organization estimated that 40.3 million individuals were subject to exploitation, including human trafficking, with 71% of the victims being women [8]. Survivors of sexual assault face heightened risks of various adverse health outcomes, including complications in reproductive health, behavioural changes, mental health disorders, and, in severe cases, fatal outcomes [9]. Consequently, multidisciplinary and immediate care is imperative in cases of sexual assault, with the objective of guaranteeing that the professionals involved are fully aware of their responsibilities with respect to the victim. In some countries, including the United States and a number of European nations, specialist centres employ the services of sexual assault nurse examiners (SANEs) and sexual assault response teams (SARTs) [10].

In the 1970s, sexual assault response teams (SARTs) were established with the objective of training professionals in the provision of assistance to victims of sexual assault. Lynch, in the 1990s, described the role of forensic nursing in working with trauma victims [11]. The teams employ a comprehensive range of resources, maintaining a non-judgmental approach throughout. The teams comprise healthcare professionals (SANEs, forensic doctors, or both, according to the hospital in question), police officers, advocates from rape crisis centres, and prosecutors. These centres are operational on a 24 h basis, with each professional assuming a specific role, thereby necessitating effective teamwork. The team members convene on a regular basis to disseminate information obtained during the course of the victims’ treatment and recovery. In these meetings, the role of the victim advocate is to serve as a spokesperson, thereby obviating the necessity for the victim to recount their experience to each member of the SART team [10].

Sexual assault nurse examiners (SANEs) emerged along with the SART teams, in response to complaints from victims about the insensitive treatment in emergency departments [12]. There are US protocols where SANE programmes have been developed [13]. To become a SANE, nurses must be certified, possess at least two years of professional experience, and complete a rigorous didactic training programme ranging from 40 to 96 h, including theoretical and practical components depending on the location. SANEs can work in prisons, hospitals, emergency departments, forensic clinics, psychiatric centres, and universities [12]. The work of SANE nurses is based on the principles of trauma-informed care (TIC), which entails a recognition of the multifaceted nature of trauma experienced by victims. This approach aims to mitigate the adverse health impacts associated with trauma and facilitate access to additional support for healing [14]. Their services are tailored to meet the specific needs of the individual, ensuring that the victim is not retraumatised during the process. A key aspect of this approach is creating a “safe space” for the patient [15]. SANE nurses’ responsibilities include conducting a physical examination while explaining each step, collecting forensic evidence, testifying in court, and providing education and planning for sexually transmitted infections and pregnancy. Additionally, they offer emotional and social support throughout the process in collaboration with rape crisis advocates. In cases of severe injuries like internal bleeding, wounds requiring sutures, or broken bones, SANEs delegate the treatment to emergency department nurses [16]. Thus, SANE programmes are effective in facilitating psychological recovery, providing comprehensive care, collecting forensic documentation and evidence, and enhancing prosecution and community change [17].

Given the rising prevalence of sexual violence and the absence of standardised protocols across Spain’s autonomous communities, exploring the internal practices in victim care is essential. Understanding how SANEs operate in different healthcare systems can offer valuable insights into improving care quality and addressing gaps in the current approaches.

The primary aim of this study was to synthesise and describe the existing literature on the satisfaction levels of both patients and healthcare professionals regarding the role of sexual assault nurse examiners (SANEs). The specific objectives included:

- Describing the benefits of implementing SANEs in hospitals and clinical settings;
- Determining the importance of specialised education for emergency department nurses managing sexual violence cases.

## 2. Materials and Methods

To address the stated objectives, a systematic literature review of the literature was conducted. This review followed the Preferred Reporting Items for Systematic Reviews and Meta-Analyses (PRISMA) methodology [18]. A protocol was registered with the Open Software Foundation (OSF) (https://osf.io/g95zc/) (accessed on 21 November 2024).

### 2.1. Search Strategy

The PICO (population, intervention, comparison, and outcome) strategy [19] was formulated as follows: P, victims of sexual violence and the healthcare professionals involved in their care; I, the presence and implementation of SANEs; C, care without the involvement of SANEs; O, satisfaction levels of victims and healthcare professionals regarding the quality of care and the management of sexual violence cases. The search for articles was performed between November 2023 and January 2024, utilising the PubMed, Web of Science, and Scopus databases.

### 2.2. Search

The following keywords were selected for the search strategy: the DECS and MeSH terms “sexual assault”, along with the free terms “SANE” and “sexual assault nurse examiners”. These terms were combined using the Boolean operators OR and AND, resulting in the following search equation: (SANE OR “sexual assault nurse examiners”) AND “sexual assault”.

### 2.3. Study Selection

The studies retrieved from the search were imported into the Mendeley reference manager, which facilitated the removal of duplicate entries. The studies underwent independent screening by two reviewers, A.F.-C. and M.C.M.-F. Any conflicts were resolved through discussion.

The search and subsequent selection of studies was based on the following inclusion and exclusion criteria, which are outlined below.

Inclusion criteria:

- Publication date: Studies published between 2013 and 2023;
- Focus on satisfaction: Studies reporting data on the satisfaction levels of users (patients) and professionals with SANEs’ services;
- Impact evaluation: Studies analysing the needs of emergency departments and the effects of educational interventions by SANEs on user care;
- Population: Studies in with a sample consisting of adult women.

Exclusion Criteria:

- Irrelevant topics: Studies with titles including the terms such as: “children”, “paediatrics”, “adolescents”, “telemedicine”, “mental illness”, or “criminal justice”;
- Study type: Systematic reviews and articles primarily focused on SANEs’ burnout or their roles in rural areas;
- Relevance: Studies that did not provide information directly related to the objective of this work.

### 2.4. Analysis of the Risk of Bias

To guarantee a comprehensive and objective assessment of the included studies, two distinct quality evaluation frameworks were utilised, tailored to align with the specific methodological approaches of the studies in question. The quality of qualitative studies was assessed using Sarah J. Tracy’s Eight Big-Tent Criteria for Excellent in Qualitative Research [20]. Each study was evaluated systematically on the basis of eight quality dimensions: worthy topic, rich rigor, sincerity, credibility, resonance, significant contribution, ethical considerations, and meaningful coherence. For quantitative studies, the Joanna Briggs Institute (JBI) Critical Appraisal Checklist was applied [21]. This framework includes specific questions designed to assess internal validity (e.g., selection bias, measurement accuracy) and external validity (e.g., generalisability of findings).

## 3. Results

**Figure 1:**
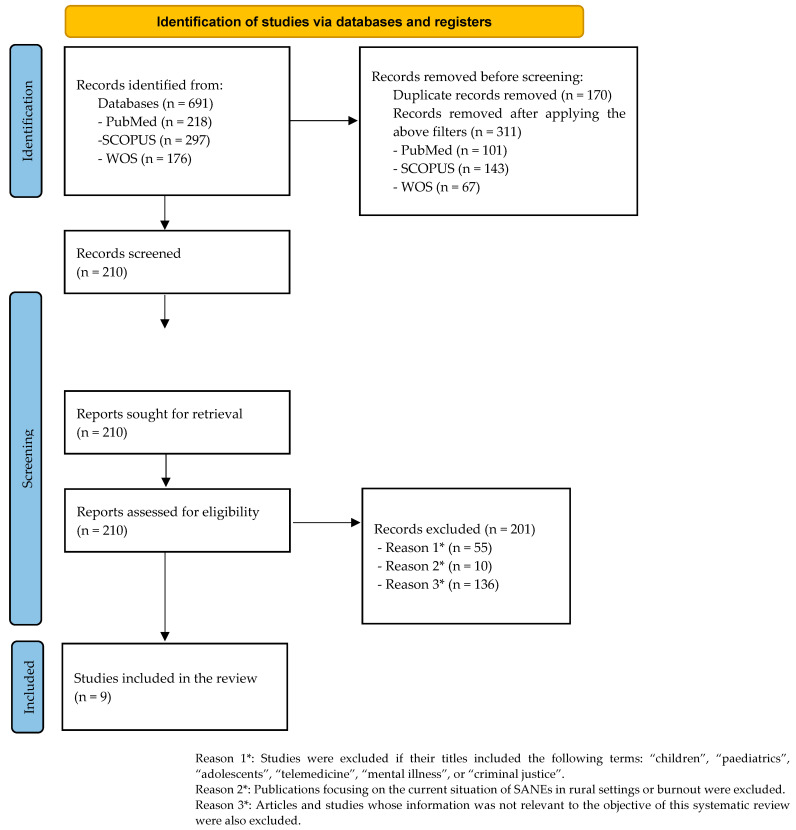
PRISMA flow diagram.

Initially, 691 articles were obtained; following application of the described inclusion criteria, 210 relevant articles were identified. After reviewing the abstracts and results and applying the exclusion criteria, the total was reduced to nine, as shown in the flowchart (Figure 1). The data extracted from each article are organised in Table 1, which includes relevant information for each study. This table contains the title, authors, and publication date; the study’s objective; the type of study and the methodology used; and the key results. Additionally, the quality of the selected studies has been analysed (Table 2 and Table 3).

Regarding the country of publication, in instances where it was not explicitly delineated in the methodology, the country of publication of the authors was incorporated: 77.8% of the studies included in this review were conducted in the United States [22,23,24,25,26,27,28], while 11.1% were conducted in Canada [29] and another 11.1% in England [30].

In terms of the methodology used in each study, 11% were retrospective (n = 1) [29]; 11% were large-scale longitudinal cohort studies (n = 1) (19); 44% were cross-sectional studies (n = 4) [22,24,25,26]; 11% were observational, longitudinal, and prospective studies (n = 1) [27]; 11% were qualitative studies (n = 1) [30]; and the final 11% were quantitative descriptive exploratory cross-sectional studies (n = 1) [28]. Furthermore, all studies utilised surveys for data collection, with 55% focusing on the opinions of healthcare professionals and 44% targeting the experiences of sexual assault victims.

**Table 1:** Results.

Author, Year and Citation	Aim	Methodology	Results
Patient satisfaction			
Du Mont J., 2014 [29]	To evaluate the satisfaction of individuals treated by SANEs in the emergency services of Ontario.	Retrospective study of 1484 women who attended 35 SANE services (n = 1484), 96% in Canada. Satisfaction survey using Likert scale.	- Care was provided in a sensitive manner (95.4%). - They felt safe during the visit (95.3%). - All their questions were answered (95.3%). - They were able to choose the care they preferred (94.8%). - The time spent with them was sufficient (94.7%). - They were treated with respect (94.8%). - The information they received was satisfactory (94.4%). - They would recommend the SANE service to a friend or family member (95.3%). - They did not feel judged (95.3%). - Care was good or excellent (98.8%). Dissatisfied users complained about long waiting times (38.2% waited more than 60 min) and the assistance provided by the emergency staff (non-SANEs).
Buchbinder M., 2021 [27]	To explore the post-discharge experiences of women who are survivors of sexual assault and to identify the specific needs that healthcare professionals should address to improve their care and support.	Observational longitudinal prospective study (n = 706) in US.Participants were adult women who received SANE care within 72 h following a sexual assault. Participants were asked the same question at three time points: “What do you believe is most important for researchers to understand about your experience since the assault?” The responses were collected one week, six weeks, and one year post-assault.	Challenges in the year following the assault included mental distress, altered self-perception, financial difficulties, struggles with intimate relationships, isolation, anxiety, and barriers to accessing healthcare. The most frequently mentioned themes were mental health (n = 401), including anxiety (n = 64), depression (n = 40), and post-traumatic stress disorder (PTSD) (n = 14). Recovery (n = 332) was also a significant theme, with difficulties in relationships and decreased income reported. For those who received counselling and support, the percentage of recovery increased (n = 112), whereas some women expressed that they “will never recover” (n = 39). A significant number of participants did not revisit discussions about the assault due to a lack of support or because it was too painful (n = 76), which diminished their hope for recovery. During the first week, participants agreed that the assault impacted all aspects of their lives (n = 55) and that they felt shattered and worthless (n = 28). They encountered barriers to accessing medical services, including high costs, difficulties identifying the necessary services, long waitlists, and emotional challenges in continuing follow-up care after the assault. For instance, some expressed, “I feel anxious thinking about calling the psychiatrist”, and noted being treated insensitively, stating, “There was little support and communication during testing for diseases, etc., after the assault” (n = 102). SANE services provide quality of care (n = 24). On the basis of these results, it was concluded that healthcare professionals play a crucial role in the long-term recovery from sexual assault trauma, and that there are numerous deficiencies in the care provided that need to be addressed.
Lechner, 2021 [23]	To explore patients’ opinions regarding the services provided by SANEs	A large-scale longitudinal cohort study, part of the “Women’s Health Study”, involving 695 women treated in 13 SANE programmes in the US. The women were evaluated one week after receiving treatment in the emergency department. Two key questions were asked: To what extent did the SANEs provide high quality of care? Did patients’ perceptions of the care they received differ on the basis of their demographic characteristics or previous health status?	Women who were treated by SANEs reported the following: - 90% felt their needs and concerns were taken seriously; - 89% noted that SANEs did not act as if the assault was their fault; - 88% said SANEs demonstrated care and compassion; - 86% felt the sexual assault examination was explained well; - 75% were provided with follow-up information. The majority of women affirmed receiving high quality of care from SANEs. The quality of care did not significantly vary according to the patients’ demographic characteristics or their prior health status.
Healthcare professionals’ satisfaction			
Cowley R., 2014 [30]	To understand the role of sexual assault nurse examiners (SANEs) in England through their experiences and opinions	Qualitative study utilising semi-structured individual interviews (n = 5 SANEs) in England. Th e participants were required to have been registered examiners for at least 5 years and have completed 15 forensic medical examinations in the past 180 days.	The participants reported the following. - Their training is adequate, but each was trained differently (theoretical training varied from 1 day to 1 year; the number of examinations performed under supervision ranged from 3 to 15; only one participant was trained to care for both children and adults). - Their confidence in performing the job stems from their additional efforts (reading textbooks, visits to the Forensic Science Service, etc.) (n = 3). - The roles of nurses and SANEs often conflict: “The two roles conflict because we are trained to be compassionate, empathetic, and all that. But when you are a forensic examiner, you have to be neutral and impartial. It feels like you are going against everything you were trained for as a nurse.” - The SANE service increases the likelihood of providing comfort care to complainants, such as access to a shower (83.3%) and clean clothing (88%). - They provide prophylaxis for common STIs such as gonorrhoea, chlamydia, and HIV (n = 1). - They only provide PEP for HIV (n = 1). Each nurse provides different services, highlighting the need to establish uniform national practice standards. - Physicians do not accept the work of SANEs: “Everyone was very skeptical at first because no one had heard of SANEs” (n = 5). - Medical supervision is important when starting SANE practice. - SANEs offer better patient service, as they take a more holistic approach and spend more time with patients.
Hollender M., 2023 [22]	To assess whether the presence of SANEs in the emergency department improves the care provided to patients who have experienced sexual assault.	A cross-sectional study in US analysing encounters between emergency department staff (SANEs and non-SANEs) and sexual assault victims (n = 182) from 1 June 2019, to 30 June 2022. Key aspects examined included resources offered, clinical care data, and continuity of care post-discharge.	Patients treated by SANEs (n = 130) had a treatment abandonment rate of 2.3%, whereas those treated by a non-SANE medical/nursing team (n = 52) had a higher abandonment rate of 15.4%. The services offered included: - Sexual assault advocate support: 97.7% (SANEs) vs. 89.4% (non-SANEs); - Forensic kits offered: 100% (SANEs) vs. 93.6% (non-SANEs); - Pregnancy tests: 92.6% (SANEs) vs. 86.1% (non-SANEs); - Post-discharge resources: 69% (SANEs) vs. 48.9% (non-SANE); - Emergency contraception: 94.3% (SANEs) vs. 82.4% (non-SANE). Patient care was better received when provided by SANEs.
Benefits of SANEs			
Nielson M.H., 2015 [24]	To determine whether the care provided by emergency nurses improves after receiving training from SANEs.	A cross-sectional study was conducted using the Rape Victim Attitude Scale in US. The sample included 1,503 nurses, of whom 5.6% (n = 22) were certified SANEs, while 94.4% (n = 372) were not SANE-certified. Additionally, 17% (n = 67) of nurses had attended courses taught by SANEs. Among the respondents, 85.5% (n = 338) reported regularly attending to sexual assault victims, while 13.7% (n = 54) indicated that they did not attend to sexual assault victims.	- Victims of sexual assault treated by non-specialised nurses receive inferior care, indicating that the presence of SANEs enhances patient outcomes and improves emergency service efficiency. - SANEs exhibit a more positive attitude toward patients, while non-specialised nurses tend to harbour biases and negative attitudes. - Nurses lacking training may perceive these patients as burdensome, as they require extended periods of care (4 to 6 uninterrupted hours) and have numerous physical and psychological needs. This situation can lead to increased wait times for other patients. - Forensic education should be incorporated into nursing curricula.
Chandramani A., 2020 [25]	Evaluate and address the self-perceived educational gaps among emergency staff in Illinois by implementing an educational intervention led by SANEs.	A cross-sectional study assessed the educational needs for a SANE intervention using a Likert scale (n = 95 professionals; including n = 20 physicians, n = 34 residents, and n = 41 non-SANE nurses) in the US. Initially, a self-assessment of the emergency department staff’s perceived needs when dealing with sexual assault cases, along with their attitudes and beliefs, was conducted. Subsequently, these findings informed an intervention led by SANEs aimed at emergency medicine residents, comprising a didactic lecture on two case studies and simulations of examinations. Finally, participants completed a follow-up survey to compare their needs, beliefs, and attitudes before and after the intervention.	Before the intervention, the survey results indicated the following. - The participants felt comfortable advising on the need for HIV prophylaxis (68%), sexually transmitted infection (STI) prevention (80%), pregnancy options, and emergency contraception (72%), but only a few felt comfortable conducting forensic examinations (29%). - They felt confident in their ability to avoid retraumatising the patient (51%). - Only 20% were aware of the laws regarding evidence collection and storage during forensic examinations, while 33% understood their hospital’s protocols in these cases. - Thirty-seven percent agreed that sexual assault patients took time away from more critical patients, and 71% considered time a significant barrier to conducting thorough and sensitive forensic examinations. - Only 26% felt qualified to provide trauma-informed care. - In total, 87% expressed interest in receiving the SANE intervention, with 7 professionals indicating a need for additional SANE practitioners. - The participants generally agreed that they always believed that patients reporting sexual assault were truthful (n = 94). - In total, 67% were aware of the specific elements of the patient’s history that needed to be obtained. After the targeted intervention, participants strongly agreed about the following. - They understood the specific elements of the patient’s history that should be collected (93%). - They felt comfortable advising patients regarding the forensic examination (increased from 41% to 86%). - They felt confident in their ability to avoid retraumatising sexual assault patients through their actions (increased from 33% to 80%). - They felt comfortable using the evidence collection kit (increased from 44% to 87%). - They were aware of their hospital’s policies and protocols (increased from 17% to 73%). - The perception that these patients take time away from critical cases decreased from 56% to 40%. Some comments from the staff included the following. “I feel that I have never been formally taught how to treat a sexual assault patient.” “I don’t know how to properly conduct a SANE exam from start to finish.” “There is a need to increase the number of SANEs in Illinois and across the country to meet the care needs of survivors and the educational requirements of emergency professionals.”
Wolf L., 2022 [28]	To gather data on the knowledge and training of SANEs, identifying gaps in nursing skills and practices, thus developing appropriate education for this specialty.	Exploratory, descriptive, and cross-sectional study, aimed at emergency nurses in the US (n = 1824).	The results led to the following conclusions: - 55.9% of hospitals had SANEs’ services available; - 47.1% of professionals had treated sexual assault cases in the emergency department, but only 12.4% felt competent to provide care to these patients; - 95.4% agreed that education and training in forensic nursing are necessary for treating this type of patient; - 91.2% lacked adequate training in forensic nursing, which impacted the collection and preservation of evidence in 96.9% of cases; however, 83.2% of professionals had received some form of training in forensic nursing through continuing education programmes; - Emergency nurses did not possess sufficient competence to recognise and care for these patients, and many professionals primarily focused on physical injuries, neglecting the emotional and psychological needs of the patient
Chalmers J., 2023 [26]	Evaluate the difference in training regarding sexual assault cases between personnel with SANE certification and those without such training.	Cross-sectional study (n = 321). Electronic surveys were administered to personnel working alongside SANEs in US. The questions focused on two main topics: staff training and the improvement in quality attributed to the presence of SANEs.	The respondents agreed that: - SANEs provide trauma-informed, high-quality care; - 66.7% of hospitals are equipped with evidence collection kits; - 55.3% of SANEs are part of the healthcare team; - 53.2% of emergency department staff (non-SANEs) expressed scepticism, either verbally or non-verbally, regarding a patient’s account of a sexual assault; additionally, 28.35% blamed the survivors for the circumstances surrounding their assault, leading to retraumatising interactions; - 83.6% provided thorough explanations of all medical care, and 78.4% requested consent at each step of the examination (most cases involved a SANE); - 43.9% of emergency department staff (non-SANEs) pressured the survivors to complete the examination; - 70.7% of patients experienced long wait times; - 71.5% of survivors had to repeat their story to multiple members of the care team; - Only 65.8% of emergency department staff felt comfortable conducting forensic examinations; - 57.9% of emergency department staff had the resources to support patients after discharge; - Healthcare professionals require more robust training; moreover, the availability of SANEs should be equal across all regions, not just in major cities.

**Table 2:** Quality of qualitative studies.

Study	The Research Topic Is Relevant, Timely, and Significant	The Study Uses Sufficient and Appropriate Data and Samples	The Study is Characterised by Transparency Regarding Its Methods and Challenges	The Research is Marked by a Thorough Description of the Participants	The Research Impacts Specific Readers Through Transferable Conclusions	The Research Offers a Significant Contribution both Morally and Methodologically	The Research Considers the Ethics of the Procedures and the Context Involved	The Study Successfully Achieves Its Objectives
Cowley R. [30]	✓	✓	✓	✓	✓	✓	✓	✓

Note. The symbol ✓ indicates that the condition or criterion is met.

**Table 3:** Quality of quantitative studies.

Study	Was the Study Powerful Enough to Detect an Effect?	Is There a Risk That the Statistical Analyses Carried Out by the Study Will Reveal an Effect That Does Not Actually Exist?	Have Confounding Factors and Conflicts of Interest Been Identified and Described by the Authors?	Was an Ethics Statement Required for This Research and, If So, Was It Provided?	Was True Randomisation Used for the Allocation of Participants to Treatment Groups?	Was an Appropriate Statistical Analysis Used?	Were the Study Subjects and the Environment Described in Detail?	Are the Results Generalisable to Groups, Populations or Contexts That Did Not Participate in the Study?
Hollender M. [22]	✓	✗	✓	✓	✓	✓	✓	✓
Lechner M. [23]	✓	✗	✓	✓	✓	✓	✓	✓
Du Mont J. [29]	✓	✗	✗	✗	✓	✓	✓	✓
Nielson M.H. [24]	✓	✗	✓	✓	✓	✗	✓	✓
Chandramani A. [25]	✓	✓	✓	✓	✓	✓	✓	✓
Chalmers J. [26]	✓	✗	✓	✓	✓	✓	✓	✓
Buchbinder M. [27]	✓	✓	✓	✓	✓	✗	✓	✓
Wolf L.A. [28]	✓	✗	✓	✓	✓	✓	✓	✓

Note. The symbol ✓ indicates that the condition or criterion is met, while the symbol ✗ indicates that the condition or criterion is not met.

### Quality of the Articles

The quality assessment of the included studies can be found in Table 2 and Table 3. Table 2 provides a summary of the evaluation of the qualitative studies, while Table 3 presents the results of the quality assessment for the quantitative studies.

## 4. Discussion

The aim of this paper was to provide a comprehensive account of the extant literature pertaining to patient and professional satisfaction with sexual assault nurse examiners (SANEs). In order to achieve this, it was necessary to conduct a systematic review of recent literature addressing patient and professional satisfaction with the work of SANEs. The results of this review are based on a total of nine studies, all of which underwent a rigorous quality assessment. These studies demonstrated strong scientific rigor, with high-quality designs and methodologies, which bolsters the overall scientific strength of the evidence. Following the analysis of the collected data, this discussion section addresses three key points: patient and staff satisfaction, the importance of SANE personnel, and the benefits of their implementation in hospitals, along with the necessity for specialised education.

The level of patient satisfaction is directly proportional to the quality of care provided by healthcare professionals. It is therefore imperative to conduct further research on this topic, as there may be a necessity to enhance the training of professionals [24]. In terms of patient satisfaction, the findings indicate that SANEs are mindful of the needs and concerns of victims, refrain from blaming them (thereby avoiding retraumatisation), and maintain a compassionate and careful attitude. Patients describe the practice environment as one where they feel safe and secure [23,29]. Moreover, they elucidate the examination process in a step-by-step manner, furnish follow-up information, engage in discourse regarding the full range of available treatment options with the woman, and give due consideration to her preferences [29]. The literature concurs on the level of satisfaction with SANEs, with 95.3% of participants indicating a willingness to recommend them to a friend or family member [22,29]. In terms of treatment, the study by Hollender asserts that patients are more likely to accept a greater number of techniques and forms of assistance when attended by SANEs [22]. For instance, 88.4% of patients consented to the use of the forensic kit by SANEs, compared with 68.2% who consented when treated by non-specialised staff [22]. Similarly in another study, adherence to the established treatment plan and attendance at a social worker’s appointment was 100% for patients attended by specialised nurses versus 84.6% for those treated by non-SANE personnel [23]. This may be attributed to the fact that women attended by SANEs reported receiving quality of care. Indeed, 90% of respondents felt that their concerns and needs were heard and addressed, 89% did not feel judged, and in 86% of cases, the professionals explained all the steps of the examination [23].

With regard to patient dissatisfaction with the SANE team, this is primarily attributable to the lengthy waiting periods (38.2% of patients waited for over 60 min) [29]. In addition, another study observed wait times of up to nine hours, with a considerable number of tests raising concerns about their validity [26]. Furthermore, discontent with the quality of their work has been identified. In the article by Fehler-Cabal, a sexual assault victim stated that while the SANE was pleasant, she followed a routine and seemed eager to conclude the examination as quickly as possible, without consideration for the patient’s emotional state [31]. Additionally, other victims have reported that their nurses were unresponsive, detached, and did not provide explanations regarding the examination process or address their inquiries. Furthermore, they did not offer options for the examination procedures or demonstrate the evidence discovered (such as bruises or scratches) [27]. Conversely, the study by Nielson indicated that patient satisfaction declines when non-specialised professionals are involved in the care of patients with sexual assault injuries. These same professionals have been known to view these users as a burden, citing the extended period of time required for their care due to the physical and psychological injuries sustained [24,25]. In this regard, it has been observed that only 29% of non-SANE staff feel comfortable attending to these users, and only 33% are familiar with the protocol in these cases. Furthermore, the quality of care provided by non-SANE personnel is inferior, as evidenced by Chalmers’ findings. Specifically, 53.2% of users reported feeling judged due to the scepticism displayed by emergency staff, while 28.35% experienced insinuations that they were somehow responsible for the circumstances surrounding the sexual assault [26].

In terms of the advantages afforded by SANEs, the presence of such personnel within a hospital setting has been shown to engender a sense of enhanced comfort and security for both patients and professionals alike [21,23]. In the study conducted by Chandramani, a survey was administered to professionals prior to and following the receipt of training provided by SANEs. One outcome that illustrates enhanced security for personnel when SANEs are present in the hospital setting is that only 29% of non-specialised healthcare workers who attended to the users reported feeling at ease in conducting sexual assault examinations. Of this cohort, only 26% were able to provide informed care regarding trauma. However, following training from specialist nurses, these figures rose significantly to 73% and 80%, respectively [25]. The evidence indicates that SANEs provide quality of care that is focused on trauma, and that they thoroughly explain all treatments and request consent at each step of the examination in 83.6% of cases [26].

The final point of this discussion is to consider the necessity of specialised training for those caring for victims of sexual assault. This is a need that is mentioned in studies directed at SANE personnel. The initial study to highlight this necessity was conducted by Chalmers [26], who surveyed respondents and found that they agreed that healthcare providers should be better prepared with more comprehensive training. Similarly, in the study by Wolf, non-specialised professionals in the emergency department conceded that they lacked the requisite competence to attend to these users, which affected the collection and preservation of evidence in 96.9% of cases. Furthermore, in many cases, the focus is primarily on the physical injuries, with insufficient attention paid to the emotional and psychological needs of the patient [28]. However, deficiencies and differences in training are also evident among SANEs. In the study by Cowley, five SANEs reported that the duration of the theoretical training period varied considerably, ranging from one day to one year. The practical training period also exhibited significant variation, with a range of 3 to 15 supervised examinations. Moreover, they have had to undertake additional training, such as reading books or visiting the Forensic Science Service [30]. Although this figure is not defined in many countries, studies are being conducted to address this gap in the literature. For example, a study was recently conducted in Brazil on SANE training with US nurses. Despite the limited sample size of 20 nurses in a five-day training period, the study demonstrated that the nurses were able to consolidate important knowledge for professionals in charge of victim care [32].

In terms of the care they provide, it is notable that not all SANEs adhere to each step of the national guidelines for the treatment of sexual assault patients. Additionally, the provision of prophylaxis for sexually transmitted infections (STIs), such as gonorrhoea or chlamydia, is not universal. Rather, it is most commonly offered for HIV [30]. Moreover, all the women in this study concurred that assuming the role of a SANE compels them to feel they are failing to fulfil their obligations as nurses, as they are required to maintain neutrality and impartiality, which necessitates the suspension of empathy [30]. A further shortcoming of SANEs’ training is the stigmatisation of obesity during forensic examinations. Women with overweight and obesity encounter difficulties during care, including the lack of examination tables of an appropriate size, which may compromise their safety when getting on and off them, the absence of adequately sized vaginal speculums in the sexual assault kit, and the use of gowns that are too small, which may contribute to the stigmatisation of women with obesity [33]. As previously stated, the focus of SANE care is on the trauma experienced by the victim. It is therefore recommended that these patients be informed that additional materials, light sources, or staff may be required to assist them in maintaining certain positions prior to the examination, thus preventing the stigmatisation that these patients often experience.

Furthermore, another issue that requires attention is the long-term efficacy of SANEs’ work [27]. As posited by Buchbinder et al., it can be surmised that SANEs’ work is more efficacious in the short term. Over the course of weeks, most patients exhibit heightened anxiety, distress, and physical and emotional discomfort, and a deterioration in their economic and personal relationships. This is because a considerable number of study participants were unable to complete follow-ups with the relevant specialist staff members as a result of economic constraints or anxiety about attending a session with a psychologist [27]. It can therefore be surmised that comprehensive training is of paramount importance, although the shortcomings previously outlined must be rectified. Therefore, there is a need for individualised plans to address training, staffing, and the SANEs’ support platform according to each jurisdiction and hospital system [14].

There are several limitations in this systematic review that must be acknowledged. First, few countries worldwide have conducted research to assess the satisfaction of sexual assault victims with emergency nurses. Consequently, most of the studies utilised were from the United States. Additionally, there is a scarcity of research focusing on the satisfaction of both patients and healthcare personnel with SANEs, resulting in a limited selection of articles for analysis.

## 5. Conclusions

The findings of this review highlight the importance of integrating sexual assault nurse examiners (SANEs) into emergency care systems, particularly in hospitals. These specialised professionals are of paramount importance in providing victims with safe and high-quality care, while minimising the risk of retraumatisation. It can be observed that patients express a high level of satisfaction with the work of SANEs. The evidence indicates that patients feel more at ease when attended to by SANEs, as they do not typically experience feelings of being judged or pressured, which contributes to a more expeditious and less traumatic recovery. In light of these findings, it is recommended that emergency nurses receive training in SANE practices. Furthermore, additional research should be conducted to gather further data on the work of this specialised personnel and the satisfaction levels of patients.

Although relatively unknown in many countries, the role of forensic sexual assault nurse examiners is important for the provision of quality care to victims of sexual assault and should be integrated into emergency departments in a variety of healthcare settings. To this end, policymakers could establish standardised protocols and ensure that the necessary resources are available. This would improve and strengthen the provision of healthcare to victims. Furthermore, there is a necessity to develop training programmes for healthcare workers in general, with particular emphasis on those employed in hospital emergency departments. These programmes need to include detailed information regarding the treatment of sexual assault victims, delivered at a technical level, in addition to the provision of exemplary care. Finally, this review emphasises the importance of conducting further research into patient satisfaction and the recovery process associated with care provided by SART and SANE teams.

## Data Availability

Data are contained within the article.
